# Cooling extender choice modulates the efficacy of freezing extenders for boar sperm cryopreservation

**DOI:** 10.1590/1984-3143-AR2025-0174

**Published:** 2026-07-20

**Authors:** Nina Miglioranza Vellozo, Gabriel Augusto Monteiro, Pedro Henrique de Oliveira Baldini, Rogério Araújo de Almeida, José Antônio Dell’Aqua, Frederico Ozanam Papa, Daniella Jorge de Moura, Eunice Oba

**Affiliations:** 1 Departamento de Cirurgia Veterinária e Reprodução Animal, Faculdade de Medicina Veterinária e Zootecnia, Universidade Estadual Paulista – UNESP, Botucatu, SP, Brasil; 2 Faculdade de Engenharia Agrícola, Universidade Estadual de Campinas – Unicamp, Campinas, SP, Brasil

**Keywords:** boar semen, cryopreservation, cooling extender, extender interaction, cryotolerance

## Abstract

Boar semen cryopreservation is limited by high sperm sensitivity, with the cooling extender and holding time representing critical steps. However, the interplay between transport and freezing extenders remains poorly elucidated. This study evaluated the main effects and the interaction between two cooling extender (BTS and BSUI®) and two freezing extenders (TRIS® and BBB®) on post-thaw boar semen quality. Ejaculates from 10 boars were processed in a 2x2 factorial design (BTS/TRIS, BTS/BBB, BSUI/TRIS, BSUI/BBB). Sperm kinetics (CASA) and plasma membrane integrity (PMI) were assessed at pre-centrifugation, post-centrifugation, and post-thaw time points. At the post-thaw evaluation, no significant interaction was observed between transport and freezing extenders (P > 0.05), indicating that these factors act independently. Regarding the main effects, the BSUI® cooling extender resulted in superior kinetic performance compared to BTS (P < 0.05). Independently, the TRIS® freezing extender yielded higher motility and membrane integrity than BBB® (P < 0.05). Consequently, the sequential combination of BSUI/TRIS presented the highest absolute kinetic values (Total Motility 63.3%; Progressive Motility 36.1%) due to the cumulative effect of the most efficient extenders. In conclusion, the cooling extender influences post-thaw quality independently of the freezing extender. The BSUI/TRIS sequence optimized post-thaw kinetics through an additive effect. Therefore, protocol optimization should focus on selecting the most efficient extender for each isolated stage of the process.

## Introduction

Boar semen cryopreservation is an essential reproductive biotechnology for the swine industry, allowing the conservation and dissemination of high-value genetic material, facilitating international transport, and contributing to herd sanitary control (Bailey et al., 2008; Yeste et al., 2017). Despite these benefits, its large-scale application remains restricted due to the high sensitivity of boar spermatozoa to the freezing and thawing process, which results in reduced motility, loss of membrane integrity, and decreased fertility when compared to refrigerated semen (Oldenhof et al., 2021; Whaley et al., 2021).

The high sensitivity of boar spermatozoa to cold shock is largely attributed to the distinct lipid composition of its plasma membrane compared to other mammals, characterized by a low cholesterol:phospholipid ratio (Bailey et al., 2008; Yeste et al., 2017). During cooling, a lipid phase transition occurs, which destabilizes the phospholipid bilayer and increases its permeability, leading to structural and functional damage (Malcervelli et al., 2020). Concurrently, the freezing-thawing process induces a marked increase in the generation of reactive oxygen species (ROS), resulting in oxidative stress, lipid peroxidation, and sperm DNA damage, factors directly linked to the loss of motility and compromised mitochondrial function (Li et al., 2018; Zhang et al., 2021).

To mitigate these damages, freezing extenders are formulated with permeable cryoprotectants (CPAs), such as glycerol, and non-permeable ones (Oldenhof et al., 2021). Historically, egg yolk is the primary non-permeable component used in boar semen cryopreservation (Orrego et al., 2019). It is believed that the protective fraction of egg yolk is the low-density lipoprotein (LDL) (Anton, 2013; Souza et al., 2015). LDLs act by stabilizing the sperm membrane during thermal stress, interacting with seminal plasma proteins, and preventing the excessive efflux of cholesterol and phospholipids (Orrego et al., 2019; Souza et al., 2015). However, egg yolk–based extenders are not chemically equivalent. Differences in buffering systems, ionic strength, and the presence of additional low-molecular-weight solutes can change how LDL interacts with sperm membranes and, consequently, how cells tolerate cooling and subsequent exposure to permeating cryoprotectants (Anton, 2013).

In addition to extender composition, semen processing steps strongly influence boar sperm cryotolerance. Boar spermatozoa should not be frozen immediately after collection and usually require a stabilization period (holding time) at 15–17 °C prior to freezing. During this phase, an adaptive interaction occurs between spermatozoa, seminal plasma, and the cooling extender (Waberski et al., 1994; Vadnais & Roberts, 2007; Yeste, 2015, 2016). Short-term cooling extenders are critical for preliminary sample management (Santos et al., 2025). Classic formulations, such as the Beltsville Thawing Solution (BTS), function as chemically defined glucose–salt systems containing chelators (EDTA) (Dubé et al., 2004). Similarly, BSUI® acts as a short-term extender composed of sugars, amino acids, and buffer solutions. These media interact with the spermatozoa to maintain cellular viability and membrane stability until the addition of specific cryopreservation diluents prior to freezing (Torres et al., 2019).

Most freezing protocols involve transporting the sample, centrifugation, and resuspension in a freezing extender (Oldenhof et al., 2021; Schäfer et al., 2017). Like transport media, freezing extenders also vary biochemically. While standard protocols often rely on TRIS-citric acid buffering systems to maintain pH during severe temperature drops, alternative proprietary formulations may utilize different buffer combinations, amino acids, and specific egg-yolk concentrations to optimize osmotic balance and cryoprotection. Because these extenders differ in buffering capacity, ionic composition, and availability of exogenous lipids, the physicochemical environment established during transport (e.g., with or without lipids) may influence the subsequent response to centrifugation and the interaction with the freezing medium. Such procedures create an opportunity for interaction effects between the cooling extender and the freezing extender, which can impact post-thaw sperm quality.

Therefore, the hypothesis of this study is that the specific combination between transport and freezing extenders interdependently affects post-thaw sperm quality. Thus, the objective was to evaluate the main effects and the interaction between two transport/refrigeration extenders, Beltsville Thawing Solution (BTS) and a sugars-based extender (BSUI®), and two freezing extenders, a Tris-egg yolk-based extender (TRIS®) and an egg yolk-based extender (BBB®), on the viability and sperm kinetics parameters of boar semen after thawing. Understanding this interaction may provide new insights for the optimization of cryopreservation protocols and for the advancement of reproductive biotechnologies applied to the swine industry.

## Methods

This project was conducted following the ethical guidelines recommended by the Brazilian College of Animal Experimentation (COBEA) and was approved by the Animal Use Ethics Committee of the School of Veterinary Medicine and Animal Science, UNESP, Botucatu (Protocol No. 108/2008).

### Study site and experimental animals

The study was conducted at a commercial swine farm located in Capivari, São Paulo, Brazil (latitude 22º59'42" S, longitude 47º30'28" W, at an altitude of 636 meters). The study was carried out during the months of January and February.

Ten hybrid commercial line boars (AG337), with a mean age of 2 years and weighing approximately 350 kg, were employed in this study. All animals were considered reproductively sound based on established standards (CBRA, 2013) and clinically healthy. The boars were housed in individual 9 m^2^ pens with concrete floors, equipped with a feed trough and a nipple drinker. The facility was covered and equipped with fans to mitigate thermal stress on hotter days. The diet provided was balanced according to the requirements of the genetic line and produced on-farm, with its nutritional composition kept constant throughout the experimental period. The production unit operated under a strict biosecurity program for access control and disease prevention in the herd.

### Extenders composition

Two cooling extenders and two freezing extenders were evaluated in this study. Regarding the transport media, the Beltsville Thawing Solution (BTS) was used as a commercial glucose-based control, acquired from IMV Technologies (L'Aigle, France) and prepared according to the manufacturer’s instructions. The second transport medium was BSUI® (Botupharma, Botucatu, Brazil), a short-term extender based on sugars, amino acids, and buffer solutions.

Regarding the freezing extenders, the TRIS extender (Tris-Egg Yolk) consisted of a buffered salt-based solution composed of 3.028 g Tris-hydroxymethyl-aminomethane, 1.78 g citric acid monohydrate, and 1.25 g D-fructose dissolved in 100 mL of ultrapure water. This base solution was supplemented with 20% (v/v) centrifuged egg yolk, 2% (v/v) glycerol, and 2% (v/v) methylformamide. The second freezing medium, BBB® (Botupharma, Botucatu, Brazil), is a commercial extender based on sugars, amino acids, and buffers. Following the manufacturer's recommendations for cryopreservation, this medium was also supplemented with 20% (v/v) egg yolk, 2% (v/v) glycerol, and 2% (v/v) methylformamide.

### Experimental design

The study was designed in a 2x2 factorial arrangement with the objective of investigating the main effects and the interaction between two refrigeration and cooling extenders (BTS and BSUI®) and two cryopreservation extenders (TRIS® and BBB®). This design allowed for the evaluation of the influence of each medium individually, as well as the synergistic effect of the combinations on sperm viability and kinetics parameters.

Therefore, ejaculates were collected and immediately diluted in the refrigeration media (BTS or BSUI®) to be transported for a period of 3 to 4 hours to the analysis site. Following transport, the samples were processed and resuspended in the freezing media (TRIS® or BBB®), thus creating the four experimental treatment groups: BTS/TRIS (transported in BTS and frozen in TRIS®), BTS/BBB (transported in BTS and frozen in BBB®), BSUI/TRIS (transported in BSUI® and frozen in TRIS®), and BSUI/BBB (transported in BSUI® and frozen in BBB®).

To isolate the effect of each protocol step, sperm quality was assessed at three critical time points. The first evaluation (pre-centrifugation) was performed after transport and warming, with the objective of establishing baseline seminal quality and isolating the effect of the refrigeration extenders during transport. The second evaluation (post-centrifugation) occurred after centrifugation and resuspension in the freezing extenders, aiming to measure the immediate impact of osmotic stress and cryoprotectant toxicity before the cooling curve. Finally, the third evaluation (post-thaw) was the final analysis after thawing, designated to quantify the cumulative damage of the entire cryopreservation process and to determine the final performance of each extender combination.

**Figure 1:**
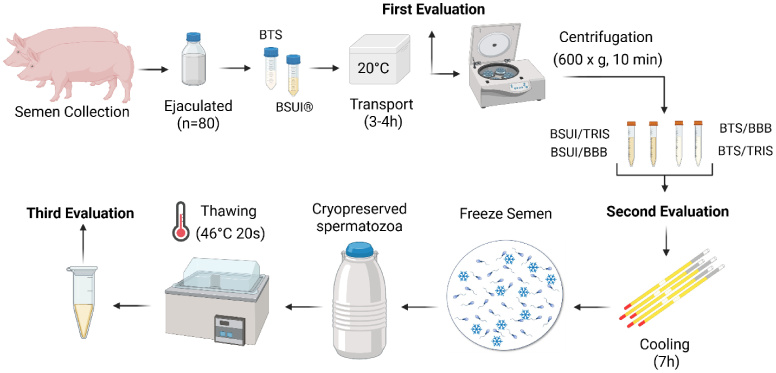
Experimental design. Flowchart illustrating the 2x2 factorial arrangement. Eighty ejaculates (n=80) were collected and divided into two cooling extenders (BTS and BSUI®), transported at 20°C (3-4h), and subjected to the Pre-Centrifugation Evaluation (1st evaluation). Subsequently, samples were centrifuged (600 x g, 10 min) and resuspended in the freezing extenders, forming the four treatment groups (BTS/TRIS, BTS/BBB, BSUI/TRIS, BSUI/BBB). At this point, the Post-Centrifugation Evaluation (2nd evaluation) was performed. Samples were loaded, subjected to the cooling curve (7h), and cryopreserved in liquid nitrogen (N_2_). After thawing (46°C for 20s), the Post-Thaw Evaluation (3rd evaluation) was conducted. Created in BioRender.

The complete flowchart, detailing the group division and evaluation points, is schematically represented in Figure 1.

### Ejaculate collection

Semen samples were collected weekly from each boar, totaling eight ejaculates per animal. Collections were performed using a dummy sow and the classic gloved-hand method (Hancock & Hovell, 1959), employing both the sperm-rich and sperm-poor fractions of the ejaculate. The gelatinous fraction, originating from the bulbourethral glands, was removed with sterile gauze and discarded.

Semen was collected into a graduated glass beaker, previously warmed to 35 °C, sterilized, and kept inside an isothermal container to preserve the sample's temperature and prevent thermal shock to the sperm cells during collection.

### Sample transport

Following collection, the ejaculates were diluted at a 1:2 ratio (5 mL semen: 10 mL extender) in two different refrigeration extenders pre-warmed to 37°C: Beltsville Thawing Solution (BTS) and BSUI (Botupharma®). Immediately thereafter, they were stored in polystyrene containers at 20°C and transported to the Laboratory of the Center for Diagnosis and Biotechnology in Animal Reproduction at the School of Veterinary Medicine and Animal Science (UNESP), Botucatu campus, for subsequent evaluation. An aliquot of raw semen from each animal was reserved for the analysis of sperm membrane integrity.

### Semen processing and freezing

The boar semen was frozen according to the following protocol. Samples transported in the two extenders (BTS and BSUI®) were centrifuged at 600 x g for 10 minutes to remove seminal plasma and concentrate the spermatozoa into pellets. Following centrifugation, the supernatant was discarded, and the pellets were resuspended in two commercial egg yolk-based freezing extenders: modified TRIS® (Botupharma®) and BBB® (Botupharma®). The sperm concentration was adjusted to 100x10^6^/mL.

Spermatozoa resuspended in the two freezing extenders were evaluated again for sperm kinetics and plasma membrane integrity. The samples were then loaded into 0.5 mL French straws and sealed with polyvinyl alcohol at room temperature. Subsequently, the straws were cooled in a Botutainer® isothermal box (Botupharma®) for 3 hours, at a cooling rate of 0.1°C/minute, until they reached a temperature of 15.0°C. After this period, the samples were placed in an isothermal box (Botutainer®, Botupharma) and cooled for 4 hours at a rate of 0.05°C/minute until reaching 5.0°C. Finally, the freezing curve was performed in a polystyrene box, where the straws were held 3 cm above the liquid nitrogen vapor level at -120.0°C for 20 minutes, and subsequently plunged into liquid nitrogen, which has a temperature of -196.0°C. After freezing, the straws were stored in a cryobiological container until analyses were performed.

### Semen analysis

Prior to any evaluation, semen samples were rapidly warmed to 37°C for 10 minutes. To prevent cell overlap and ensure accurate trajectory reconstruction by the CASA system, samples were rediluted in their respective freezing extenders (TRIS® or BBB®) to an optimal concentration. After this adjustment, sperm kinetics and plasma membrane integrity were assessed. For cryopreserved samples, straws were thawed in a water bath at 46°C for 20 seconds (Dell’Aqua et al., 2001) and immediately maintained at 37°C as previously described.

Sperm kinetics analysis was performed using a computer-assisted sperm analysis (CASA) system (HTM-IVOS 12; Hamilton-Thorne Research, Danvers, MA, USA). The instrument settings were configured as follows: 45 frames acquired at 60 Hz; minimum contrast, 65; minimum cell size, 5 pixels; VAP cutoff, 20 µm/s; progressive minimum VAP cutoff, 45 µm/s; VSL cutoff, 5 µm/s; static head size, 0.60–4.90; and magnification, 1.73x. The previously homogenized material was evaluated by placing a 10 µL aliquot of semen onto a Makler chamber (Mackler Counting Chamber®, Sefi-Medical Instruments Ltd., Haifa, Israel), pre-warmed to 37°C. Five random fields were observed, with a minimum of 150 spermatozoa per field. The sperm motion parameters evaluated were: total motility (TM, %), progressive motility (PM, %), average path velocity (VAP, µm/s), straight-line velocity (VSL, µm/s), curvilinear velocity (VCL, µm/s), amplitude of lateral head displacement (ALH, µm), linearity (LIN, %), and rapid spermatozoa (RAP, %), which considers only cells exhibiting a VAP above 70 µm/s.

Sperm plasma membrane integrity was assessed according to the method described by Harrison & Vickers (1990) using the fluorescent dyes carboxyfluorescein diacetate (20 mM) and propidium iodide (7.3 mM). A 4 µL aliquot was examined between a slide and coverslip under an epifluorescence microscope at 1000× magnification, using an L3 filter set (excitation BP 450–490 nm; suppression LP 515 nm). A total of 200 cells per sample were evaluated and classified according to Monteiro et al. (2013).

## Statistical analysis

All statistical analyses were performed using SAS software (version 9.4; SAS Institute Inc., Cary, NC, USA). The ejaculate was defined as the experimental unit. Data were analyzed using a General Linear Mixed Model (GLMM) via the MIXED procedure (PROC MIXED). The model included the fixed effects of the cooling extender (BTS vs. BSUI®), the freezing extender (TRIS® vs. BBB®), and their interaction. To account for the split-sample design and biological variability, the boar was included as a random effect in the model.

The normality of the residuals was verified using the Shapiro-Wilk test. When significant effects were detected, least squares means were compared using the Tukey-Kramer test. Significance was set at P < 0.05. Data are presented as mean ± standard deviation (SD). Variables that did not meet the assumptions of normality even after transformation were analyzed using the non-parametric Kruskal-Wallis test, followed by the Dunn test for multiple comparisons.

## Results

Assessment of sperm parameters at the three critical time points (pre-centrifugation, post-centrifugation, and post-thaw) revealed significant effects of the cooling extenders and the cryopreservation combinations on sperm kinetics and membrane integrity.

### Pre-centrifugation evaluation: effect of cooling extenders

At the first evaluation point, following transport (Table 1), no significant difference was observed in Total Motility (TM), Progressive Motility (PM), or Membrane Integrity (MI) between the BTS and BSUI® refrigeration extenders.

**Table 1:** Sperm parameters (mean ± standard deviation) evaluated after transport (pre-centrifugation time point), comparing the refrigeration extenders.

**Parameter**	**BTS**	**BSUI®**	**P-Value**
TM (%)	87.3 ± 6.6	87.8 ± 5.7	0.782
PM (%)	50.4 ± 12.8	45.7 ± 11.3	0.139
VAP (µm/s)	99.9 ± 13.0 ^b^	107.2 ± 15.1 ^a^	0.045
VSL (µm/s)	73.4 ± 10.2	75.5 ± 9.8	0.42
VCL (µm/s)	180.8 ± 25.7	193.7 ± 25.8	0.058
ALH (µm)	6.7 ± 1.0	7.0 ± 0.8	0.155
LIN (%)	45.6 ± 7.3	39.5 ± 4.7	0.078
RAP (%)	83.2 ± 7.5	83.9 ± 6.4	0.845
PMI (%)	81.5 ± 9.2	84.0 ± 7.8	0.277

TM: Total Motility; PM: Progressive Motility; VAP: Average Path Velocity; VSL: Straight-Line Velocity; VCL: Curvilinear Velocity; ALH: Amplitude of Lateral Head Displacement; LIN: Linearity; RAP: Rapid Spermatozoa; PMI: Plasma Membrane Integrity. Different superscript letters (ᵃ, ᵇ) within the same row indicate a statistical difference (P < 0.05).

However, the detailed analysis of sperm kinetics revealed specific differences between the extenders. The BSUI® extender promoted a significantly higher average path velocity (VAP) compared to BTS. No statistically significant differences were observed for the remaining kinetic parameters.

### Post-centrifugation evaluation: immediate effect of cryoprotectants

In the post-centrifugation evaluation (Table 2), which assesses the immediate impact of cryoprotectant addition before freezing, no difference was observed in TM, VAP, VSL, or RAP among the four groups.

**Table 2:** Sperm kinetics parameters (mean ± standard deviation) evaluated after resuspension in the freezing extenders (post-centrifugation time point).

**Parameter**	**BTS/TRIS®**	**BSUI®/TRIS®**	**BTS/BBB®**	**BSUI®/BBB®**
TM (%)	83.3 ± 8.5	84.3 ± 7.7	83.1 ± 7.5	86.3 ± 4.7
PM (%)	42.1 ± 10.8 ᵃᵇ	39.9 ± 9.9 ^b^	46.5 ± 9.5 ^a^	45.3 ± 9.0 ᵃᵇ
VAP (µm/s)	99.9 ± 9.1	99.1 ± 12.5	97.8 ± 9.8	101.6 ± 10.9
VSL (µm/s)	69.6 ± 7.3	69.7 ± 6.7	70.3 ± 6.4	71.7 ± 8.0
VCL (µm/s)	186.0 ± 14.7 ᵃ	188.1 ± 15.7 ^a^	177.9 ± 18.3 ^b^	182.9 ± 18.2 ᵃᵇ
ALH (µm)	7.2 ± 0.5 ᵃᵇ	7.4 ± 0.5 ^a^	6.9 ± 0.6 ^c^	7.0 ± 0.6 ^b^
LIN (%)	41.3 ± 17.9 ᵃᵇ	37.8 ± 2.4 ^b^	40.5 ± 3.1 ^a^	40.1 ± 3.1 ᵃᵇ
RAP (%)	80.5 ± 10.1	81.9 ± 8.9	80.2 ± 8.4	83.7 ± 5.2

TM: Total Motility; PM: Progressive Motility; VAP: Average Path Velocity; VSL: Straight-Line Velocity; VCL: Curvilinear Velocity; ALH: Amplitude of Lateral Head Displacement; LIN: Linearity; RAP: Rapid Spermatozoa. Different superscript letters (ᵃ, ᵇ) within the same row indicate a statistical difference (P < 0,05).

However, a clear effect of the freezing extender was identified on kinetic parameters. The BTS/BBB® medium exhibited higher progressive motility (PM) and linearity (LIN) compared to the other treatments (P < 0.05). Conversely, spermatozoa resuspended in TRIS® (BTS/TRIS® and BSUI/TRIS®) showed higher curvilinear velocity (VCL) and amplitude of lateral head displacement (ALH), with both being significantly higher in the BSUI/TRIS® group.

### Post-thaw evaluation: main effects

In the final evaluation after thawing (Table 3), statistical analysis revealed no significant interaction between the transport and freezing extenders for any of the kinetic or membrane integrity parameters assessed (P > 0.05). Thus, the main effects (transport and freezing effects) were analyzed independently.

**Table 3:** Sperm parameters (mean ± standard deviation) evaluated at the post-thaw time point, presenting the comparison between experimental groups and P-values for the effects of the cooling extender, freezing extender, and the interaction between them.

**Parameter**	**BTS / TRIS**	**BTS / BBB**	**BSUI / TRIS**	**BSUI / BBB**	**Transport**	**P-values Freezing**	**Interaction**
TM (%)	56.2 ± 14.3^ab^	52.4 ± 16.9^b^	63.3 ± 9.1ª	57.9 ± 13.5^ab^	0.002	0.003	0.866
PM (%)	30.6 ± 9.6^ab^	28.2 ± 11.0^b^	36.1 ± 6.3ª	31.9 ± 9.2^ab^	0.001	0.007	0.638
VAP (µm/s)	78.7 ± 7.6	76.2 ± 10.8	82.0 ± 9.0	79.0 ± 9.3	0.003	0.011	0.959
VSL (µm/s)	60.0 ± 6.3	59.0 ± 7.9	62.6 ± 7.8	60.3 ± 7.2	0.015	0.053	0.490
VCL (µm/s)	119.5 ± 10.6	115.5 ± 16.4	124.9 ± 11.9	122.3 ± 15.6	0.001	0.027	0.887
ALH (µm)	5.6 ± 0.7	5.5 ± 0.9	5.5 ± 1.0	5.7 ± 0.7	0.707	0.803	0.368
LIN (%)	52.7 ± 8.4	51.8 ± 3.1	50.7 ± 3.8	50.2 ± 2.9	0.067	0.366	0.898
RAP (%)	47.4 ± 13.6^ab^	43.0 ± 16.9^b^	54.0 ± 13.6ª	49.7 ± 13.7^ab^	0.001	0.012	0.840
PMI (%)	42.3 ± 12.5^a^	36.4 ± 11.1^ab^	37.3 ± 10.2^ab^	31.7 ± 10.3^b^	0.002	<0.001	0.957

TM: Total Motility; PM: Progressive Motility; VAP: Average Path Velocity; VSL: Straight-Line Velocity; VCL: Curvilinear Velocity; ALH: Amplitude of Lateral Head Displacement; LIN: Linearity; RAP: Rapid Spermatozoa; PMI: Plasma Membrane Integrity. Different superscript letters (ᵃ, ᵇ) within the same row indicate a statistical difference (P < 0.05).

Regarding the cooling extender, it was observed that samples transported in the BSUI® medium displayed significantly superior kinetic performance compared to those transported in the control medium (BTS). The use of BSUI resulted in higher values for Total Motility (P = 0.002), Progressive Motility (P = 0.001), and percentage of Rapid Sperm (P = 0.001). Additionally, BSUI promoted higher velocity indices (VAP, VSL, and VCL; P < 0.05). Conversely, when analyzing Plasma Membrane Integrity (PMI), the BTS extender yielded higher post-thaw rates, presenting values superior to those observed in the BSUI group (P = 0.002).

Regarding the freezing extenders, the TRIS® medium demonstrated statistical superiority over the BBB® medium. Freezing with TRIS resulted in higher rates of Total Motility (P = 0.003), Progressive Motility (P = 0.007), and Rapid Sperm (P = 0.012). Notably, TRIS was more effective in preserving cellular structure, presenting Plasma Membrane Integrity (PMI) values significantly higher than those obtained with the BBB extender (P < 0.001).

## Discussion

The present study showed that the transport/refrigeration extenders (BTS or BSUI®) and the freezing extenders (TRIS® or BBB®) influence post-thaw sperm quality in boars in an independent and additive manner, with no statistically significant interaction between them. These findings indicate that cryopreservation success does not rely on a specific interdependent pairing, but rather on the cumulative efficiency achieved at each step, reinforcing the need to optimize both transport conditions and the freezing-protection medium as isolated stages of the protocol.

The refrigeration and transport period (holding time) prior to freezing is widely recognized as a critical step for successful cryopreservation (Schäfer et al., 2017; Torres et al., 2019). During this period, a dynamic interaction occurs between the spermatozoa and the components of the medium, whether from the seminal plasma (SP) or the extender itself (Torres et al., 2019) Studies indicate that a holding time (HT) period (ranging from 10 to 24 hours) may stabilize the plasma membrane, making it less susceptible to subsequent cold shock (Juarez et al., 2011; Wasilewska & Fraser, 2017). Although the transport time in this study was relatively short (3 to 4 hours), detectable differences were already observed between the refrigeration extenders. While total motility and membrane integrity did not differ between BTS and BSUI®, the BSUI® extender promoted a superior VAP value, suggesting a positive effect on flagellar efficiency and mitochondrial functionality. This finding indicates that even in short exposure periods, the cooling extender's composition can induce distinct cellular responses, reflecting an initial physiological adaptation process that favors cryotolerance.

Freezing extenders play an essential role in protecting spermatozoa against osmotic, thermal, and oxidative damage occuring during the cryopreservation process and/or during the thawing procedure (Oldenhof et al., 2021; Whaley et al., 2021). Since both freezing extenders (TRIS® and BBB®) were supplemented with the exact same combination of cryoprotectants (2% glycerol and 2% methylformamide), the observed differences in post-thaw sperm quality cannot be attributed to the cryoprotective agents themselves. Instead, we hypothesize that the distinct base compositions of TRIS® and BBB®, specifically their buffering systems and solute profiles, create different physicochemical environments that modulate how these cryoprotectants interact with the sperm lipid bilayer. While the efficacy of combining glycerol with amides is known to vary depending on the buffering capacity and osmolarity of the specific freezing media (Arraztoa et al., 2017; Yang et al., 2016), our results indicate that the TRIS-based formulation provides a more optimal environment. This specific base likely enhances the protective interaction between the standardized cryoprotectants, egg yolk lipoproteins, and the plasma membrane, resulting in superior cellular integrity and motility compared to BBB®.

As expected due to the known cytotoxicity of cryoprotective agents (CPAs), the second evaluation, performed after centrifugation and resuspension in the freezing media, demonstrated an immediate impact on sperm kinetics prior to the cooling curve. At this point, a clear dissociation in kinetic patterns was observed; the BTS/BBB® combination better-preserved progressive motility (PM), whereas BSUI/TRIS® resulted in higher curvilinear velocity (VCL) and amplitude of lateral head displacement (ALH). Thus, the interpretation of these kinetic findings is complex, as CASA sperm parameters can sometimes be difficult to interpret directly in terms of fertility outcomes. Higher VCL and ALH values do not necessarily indicate greater vigor, but may reflect modifications in spermatozoa swimming ability. Specifically, an altered ALH reflects a modification in the spin axis, which may in turn affect the ability of the spermatozoa to move efficiently in a given complex environment, such as the female reproductive tract. This physical impairment aligns with a bovine study where Azevedo et al. (2024) demonstrated that bulls with the lowest fertility were those presenting the highest post-thaw VCL and ALH values. It is postulated that excessively high kinetic values may reflect premature hyperactivation or a state of osmotic stress, rather than healthy metabolism. Conversely, the more controlled and linear movement was observed in the BTS/BBB® group, a parameter that the BTS extender consistently demonstrated to preserve, which may indicate a more stable cellular state. The importance of this pre-freezing step is fundamental, as demonstrated by Moreira et al. (2025), in collared peccaries, where membrane integrity and mitochondrial activity in semen are direct predictive parameters of post-thaw success. Nevertheless, while these in vitro kinetic and structural elements are crucial for semen evaluation, they cannot replace in vivo fertility tests. Therefore, further studies are required to determine if the different extender combinations evaluated herein are correlated with actual fertility rates.

The main finding of this study was the absence of a statistical interaction between transport and freezing extenders for kinetic parameters, indicating that these stages act independently on post-thaw sperm quality. Although no mutual dependence between media was observed, significant main effects were detected for both factors, and the use of the BSUI® cooling extender and the TRIS® freezing extender consistently resulted in the best kinetic outcomes (TM, PM, and VAP).

These results indicate that the cooling extender exerts a determinant influence on cryotolerance, regardless of the freezing medium used in the subsequent step. This suggests that events occurring during the 3–4 h of refrigeration (holding period) are crucial for maintaining cell viability. It is widely accepted that transport media modulate the sperm membrane during this phase, promoting biochemical adjustments that affect lipid fluidity and the stability required to withstand later stressors (Juarez et al., 2011; Schäfer et al., 2017). The superiority of BSUI® over BTS in the present study suggests that its formulation provides a more favorable environment during initial cooling, potentially mitigating sublethal damage that would become evident only after thawing.

In parallel, the isolated effect of the freezing extender highlighted TRIS® as superior to BBB®, even though both are egg yolk–based. The effectiveness of these media in boar semen cryopreservation is strongly linked to the protective capacity of low-density lipoproteins (LDLs) from egg yolk against cold shock (Caamaño et al., 2021; Oldenhof et al., 2021). LDLs stabilize the phospholipid bilayer and prevent cholesterol efflux, which is critical in boar spermatozoa due to their low cholesterol:phospholipid ratio (Bailey et al., 2008; Yeste et al., 2017). Therefore, the superiority of TRIS® suggests that, despite a common base, its specific composition (e.g., buffering system and osmolarity) may potentiate egg yolk protection or provide greater extracellular stability than BBB®. Accordingly, the fact that the BSUI®/TRIS® group yielded the best results suggests an additional benefit of associating the most efficient transport medium with the most effective freezing formulation.

An important aspect to discuss is the dissociation observed between kinetic response and structural integrity. While transport in BSUI® favored the maintenance of motility and velocity indices (mitochondrial and flagellar function), plasma membrane integrity appears to respond differently to stressors at each stage. This dissociation reinforces the hypothesis that distinct cellular injury pathways are triggered independently during processing. Cooling and freezing induce oxidative stress and the generation of reactive oxygen species (ROS), which may impair the motility machinery before overt physical membrane disruption occurs (Li et al., 2018; Zhang et al., 2021).

Supporting this view, Malcervelli et al. (2020) reported that cooling to 5 °C more severely affects membrane functionality and mitochondrial potential than structural integrity. Thus, it is plausible that BSUI® provides better protection for energetic metabolism (preserving kinetics), whereas physical stabilization of the lipid bilayer may be modulated by mechanisms not linearly correlated with motility (Bailey et al., 2008; Zhang et al., 2021). This phenomenon is consistent with observations by Boni et al. (2025) in stallions, in which extender choice allowed discrimination among different aspects of individual cryotolerance.

In summary, the data indicate that successful boar semen cryopreservation depends on optimizing each stage of the process individually. Even a short transport period (3–4 h) was sufficient to affect the final outcome, supporting the concept that frozen semen quality is built cumulatively from the moment of collection. The sequential protocol using BSUI® (transport) followed by TRIS® (freezing) appears to be the most efficient alternative to maximize sperm kinetics, not through a direct interaction, but through the additive contribution of cellular protection at distinct stages of the cryogenic protocol.

## Conclusion

The present study demonstrates that the efficiency of boar semen cryopreservation is determined by the independent and cumulative effects of transport and freezing extenders, with no evidence of a direct interaction between them. The sequential BSUI®/TRIS® protocol yielded the best post-thaw kinetic performance, outperforming the other treatments in total motility, progressive motility, and velocity-related parameters. Conversely, the use of BTS during transport combined with TRIS® for freezing favored plasma membrane integrity, indicating that the cooling/transport medium can modulate distinct attributes of cellular quality (kinetics versus structural integrity). In addition, the TRIS® freezing extender was consistently superior to BBB®, regardless of the preceding cooling extender.

Therefore, protocol optimization should focus on selecting the most efficient extender for each stage individually, and the association of BSUI® (transport) with TRIS® (freezing) appears to be the most suitable strategy to maximize post-thaw kinetic functionality in artificial insemination programs.

## Data Availability

Research data is only available upon request.
